# Crystal structure of Na_2_HfSi_2_O_7_ by Rietveld refinement

**DOI:** 10.1107/S2056989016014225

**Published:** 2016-09-16

**Authors:** Nicolas Massoni, Pierrick Chevreux

**Affiliations:** a CEA, DEN, DTCD, Marcoule, BP17171, F-30207 Bagnols sur Ceze, France; b CRPG, CNRS UMR-5873, Université de Lorraine, BP 20, F-54501 Vandoeuvre les Nancy cedex, France

**Keywords:** crystal structure, powder diffraction, sodium hafnium disilicate

## Abstract

The structure of triclinic disodium hafnium disilicate, Na_2_HfSi_2_O_7_, has been determined by laboratory powder X-ray diffraction and refined by the Rietveld refinement. The structure is a framework made of alternate layers of HfO_6_ octa­hedra and SiO_4_ tetra­hedra linked by common O atoms. Sodium atoms are located in the voids of the framework, aligned into tunnels along the [010] direction. Na_2_HfSi_2_O_7_ is isostructural with the parakeldyshite Na_2_ZrSi_2_O_7_ phase.

## Abstract 2

A new sodium hafnium disilicate was detected as an major inter­mediate product of a global reaction between borosilicate glass and hafnium. The composition of this phase was determined to be Na_2_HfSi_2_O_7_ by laboratory powder diffraction and Rietveld refinement.

## Chemical context   

Laboratory work in order to explore the chemistry of compounds with radioactive elements such as actinides is difficult because of the emission of ionizing radiation. To overcome this problem, these radionuclides are often replaced by a stable element having similar properties as the radioactive element, for instance by using elements with a similar ionic radius or with the same oxidation state. Hence actinides are often replaced by neodymium, zirconium, europium, or hafnium (Ramsey *et al.*, 1995 ▸). The reactivity of uranium with an Na–Si–O glass at high temperatures was thus simulated by using hafnium instead of uranium. We have obtained samples with different phases among which was a sodium hafnium disilicate, similar to the sodium zirconium silicate already observed in a similar glass (Plaisted *et al.*, 1999 ▸). The structure of the sodium hafnium disilicate is discussed in this paper.

## Structural commentary   

**Figure 1:**
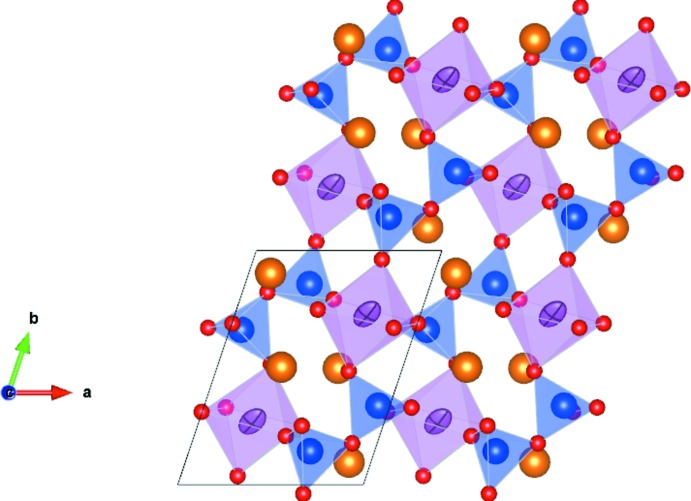
Polyhedral representation of the Na_2_HfSi_2_O_7_ phase with SiO_4_ units (blue), HfO_6_ units (green) and sodium (yellow) with displacement ellipsoids drawn at the 99% probability level.

The Na_2_HfSi_2_O_7_ phase is isostructural with the parakeldyshite phase (Voronkov *et al.*, 1970 ▸; Fleischer *et al.*, 1979 ▸). As reported in Table 1 ▸, the cell parameters of the Na_2_HfSi_2_O_7_ phase are slightly smaller than those of parakeldyshite, and the volume of the cell is 0.8% smaller. For the Na_2_HfSi_2_O_7_ phase, the Hf1O_6_ octa­hedral and the Si2O_4_ tetra­hedral volumes are about the same as the analogous Zr octa­hedral and Si tetra­hedral volumes in parakeldyshite. The Si1O_4_ tetra­hedral volume of the Na_2_HfSi_2_O_7_ phase is about 5% smaller than that in parakeldyshite. It is thus in the latter tetra­hedron that the bond lengths differ significantly whereas the other bond lengths are quite similar in both phases. The sodium coordination polyhedral volumes are quite similar in volume for the two phases, about 30.1 Å^3^. A polyhedral view of the Na_2_HfSi_2_O_7_ structure is given in Fig. 1 ▸. The Na_2_ZrSi_2_O_7_ phase is capable of ion exchange on the sodium site thanks to the sufficient dimension of the sodium tunnels in the [010] direction (Kostov-Kytin *et al.*, 2008 ▸). Since these dimensions are the same in both phases, ion exchange should also be possible in the Na_2_HfSi_2_O_7_ phase. A numerical comparison of the structures of the parakeldyshite and the Na_2_HfSi_2_O_7_ phase was performed with *COMPSTRU* (de la Flor *et al.*, 2016 ▸). The structures’ similarities were estimated by different parameters such as the measure of similarity Δ (Bergerhoff *et al.*, 1999 ▸). This parameter was determined to be 0.018 for a maximum distance between paired atoms of 1 Å, indicating that structures are effectively isostructural. Since hafnium simulates uranium, the existence of the Na_2_USi_2_O_7_ phase can also be supposed.

## Database survey   

The crystal chemistry of zirconosilicates can be described in terms of an *MT* framework with *M*O_6_ octa­hedra and *T*O_4_ tetra­hedra (*M* = Zr, *T* = Si; Ilyushin & Blatov, 2002 ▸). The voids in the *MT* framework are filled with alkaline or alkaline earth elements coordinated in an eight-vertex polyhedron. The crystal system of sodium zirconosilicates can vary from triclinic (Na_2_ZrSi_2_O_7_) to monoclinic (Na_2_ZrSi_4_O_11_) or trigonal (Na_8_ZrSi_6_O_18_). If we focus on the chemistry of zirconosilicates with Si_2_O_7_ diortho groups and their analogs (Pekov *et al.*, 2007 ▸), the triclinic phase is privileged such as the parakeldyshite Na_2_ZrSi_2_O_7_ phase (Ferreira *et al.*, 2001 ▸) or the keldyshite (Na,H)_2_ZrSi_2_O_7_ phase (Khalilov *et al.*, 1978 ▸). The potassium analogue, however, is monoclinic as in the case of khibinskite K_2_ZrSi_2_O_7_ (Chernov *et al.*, 1970 ▸; Nosyrev *et al.*, 1976 ▸).

## Synthesis and crystallization   

The synthesis of sodium hafnium disilicate was based on the two-step synthesis protocol of parakeldyshite Na_2_ZrSi_2_O_7_ (Lin *et al.*, 1999 ▸; Ferreira *et al.*, 2001 ▸). The first step was the synthesis of the Hf–petarasite phase Na_5_Zr_2_Si_6_O_18_(Cl·OH)_2_·H_2_O with zirconium totally substituted by hafnium. Adequate qu­anti­ties of sodium silicate solution (27% SiO_2_, 8% Na_2_O), sodium chloride, hafnium chloride, potassium chloride, sodium hydroxide and water were mixed thoroughly in a polytetra­fluoro­ethyl­ene (PTFE) vessel at room temperature for 30 minutes. A gel was obtained with a pH value around 13. The PTFE vessel was put in a Parr digestion apparatus for a hydro­thermal synthesis over 10 days at 523 K. The resulting powder was washed, filtered, and dried overnight at 393 K. In spite of the drying process, the powder was still hydrated. Powder X-ray diffraction showed the compound to be isostructural to petarasite. The second step was the calcination of Hf–petarasite over 15 h at 1373 K under air which lead to a white powder. SEM observation of the powder showed large grains with Na, Hf, Si and O and smaller grains with supplementary K. The chemical composition of the major phase was determined by EDS to have the following stoichiometry Na_1.7±0.2_Hf_1.0_Si_2.3±0.1_O_7.3±0.9_ as compared to the theoretical stoichiometry of Na_2_HfSi_2_O_7_. Thus the major phase is very close to the expected one. The sample was analysed by differential thermal analysis to determine its melting point. There was no thermal event indicating a melting until 1623 K and the sample was still in powder form. The Na_2_HfSi_2_O_7_ phase therefore has a higher melting point than parakeldyshite which is below 1523 K (Ferreira *et al.*, 2001 ▸).

## Refinement   

**Figure 2:**
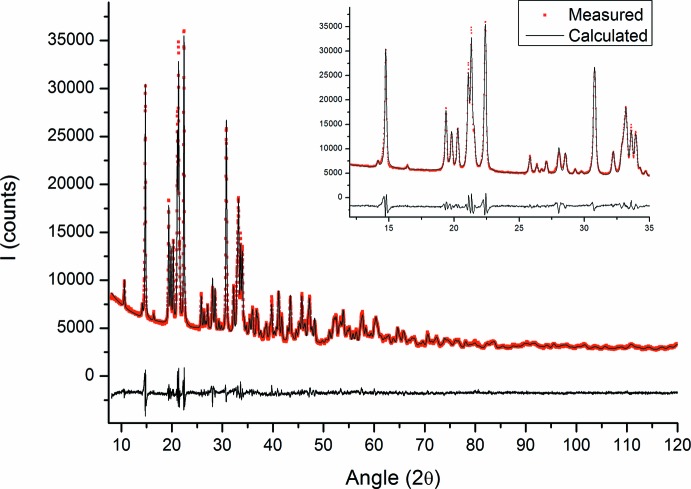
Comparison of observed (red squares) and calculated (solid line) intensities for Na_2_HfSi_2_O_7_. The difference pattern appears below. Inset: focus on the 12–35° 2θ range.

Crystal data, data collection and structure refinement details are summarized in Table 2 ▸. Observed and calculated intensities for Na_2_HfSi_2_O_7_ are shown in Fig. 2 ▸ along with the difference pattern. The reliability factors of the refinement were quite poor because of an amorphous bump attributed to the second minor phase. Hence the reliability factors were negatively impacted. The isotropic ADP’s of the oxygen atoms were constrained to be equal in volume in order to avoid a slightly negative ADP value on O5. The residual electron density is about 2.4 e Å^3^, which is less than 10% of the electron density of a Hf atom. The occupancies of all atoms were fixed to unity.

## Supplementary Material

Crystal structure: contains datablock(s) nahfsio, I. DOI: 10.1107/S2056989016014225/vn2115sup1.cif

CCDC reference: 1502957

Additional supporting information: crystallographic information; 3D view; checkCIF report

## Figures and Tables

**Table 1:** Cell parameters, selected distances (Å), angles (°) and volumes (Å^3^) for the title phase compared to parakeldyshite Cell parameters of Na_2_ZrSi_2_O_7_ are from Ferreira *et al.* (2001 ▸).

Na_2_HfSi_2_O_7_		Na_2_ZrSi_2_O_7_	
*a*, *b*, *c*	6.6123 (2), 8.7948 (3), 5.41074 (15)	*a*, *b*, *c*	6.6364 (4), 8.8120 (5), 5.4233 (3)
α, β, γ	92.603 (2), 94.084 (2), 71.326 (2)	α, β, γ	92.697 (4), 94.204 (3), 71.355 (3)
*V*_cell_	297.25 (2)	*V*_cell_	299.61 (3)
Hf1 octa­hedron		Zr octa­hedron	
O1—O7	4.15 (4)	O1—O7	4.22 (2)
O4—O5	4.28 (3)	O4—O5	4.24 (2)
O3—O6	4.22 (4)	O3—O6	4.18 (2)
Hf1—O7	2.23 (3)	Zr—O7	2.13 (2)
Hf1—O1	1.92 (3)	Zr—O1	2.08 (2)
Hf1—O3	2.30 (3)	Zr—O3	2.11 (2)
Hf1—O4	2.04 (2)	Zr—O4	2.16 (3)
Hf1—O5	2.26 (3)	Zr—O5	2.09 (3)
Hf1—O6	1.93 (2)	Zr—O6	2.03 (2)
Hf1—O7—Si2	116.7 (6)	Zr—O7—Si2	124.0 (4)
Polyhedron volume	12.5	Polyhedron volume	12.4
			
Si1 tetra­hedron		Si1 tetra­hedron	
Si1—O2^i^	1.55 (3)	Si1—O2^i^	1.62 (2)
Si1—O3^i^	1.37 (4)	Si1—O3^i^	1.58 (1)
Si1—O4^i^	1.66 (3)	Si1—O4^i^	1.55 (1)
Si1—O1	1.68 (3)	Si1—O1	1.57 (1)
Polyhedron volume	1.94	Polyhedron volume	2.02
			
Si2 tetra­hedron		Si2 tetra­hedron	
Si2—O2	1.77 (3)	Si2—O2	1.67 (2)
Si2—O7^i^	1.61 (3)	Si2—O7^i^	1.64 (1)
Si2—O5	1.61 (3)	Si2—O5	1.62 (1)
Si2—O6	1.60 (3)	Si2—O6	1.53 (1)
Polyhedron volume	2.12	Polyhedron volume	2.14
			
Si1—O—Si2 bridging angle	136.7 (5)	Si1—O—Si2 bridging angle	130.9 (5)

Symmetry code: (i) −*x*, −*y*, −*z*.

**Table 2:** Experimental details

Crystal data	
Chemical formula	Na_2_HfSi_2_O_7_
*M*_r_	392.6
Crystal system, space group	Triclinic, *P* 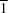
Temperature (K)	293
*a*, *b*, *c* (Å)	6.6123 (2), 8.7948 (3), 5.41074 (15)
α, β, γ (°)	92.603 (2), 94.0843 (18), 71.3262 (18)
*V* (Å^3^)	297.25 (2)
*Z*	2
Radiation type	Cu *K*α_1_, λ = 1.540562, 1.544390 Å
Specimen shape, size (mm)	Flat sheet, 25 × 25

Data collection	
Diffractometer	Panalytical XPert MPD Pro
Specimen mounting	Packed powder pellet
Data collection mode	Reflection
Scan method	Step
2θ values (°)	2θ_min_ = 8.013 2θ_max_ = 120.013 2θ_step_ = 0.017

Refinement	
*R* factors and goodness of fit	*R*_p_ = 0.024, *R*_wp_ = 0.032, *R*_exp_ = 0.015, *R*(*F*) = 0.024, χ^2^ = 4.973
No. of parameters	67

Computer programs: *X’Pert Data Collector* (PANalytical, 2011 ▸), *JANA2006* (Petříček *et al.*, 2014 ▸), *VESTA* (Momma & Izumi, 2011 ▸) and *publCIF* (Westrip, 2010 ▸).

## supplementary crystallographic information

### Crystal data

**Table:**

Na_2_HfSi_2_O_7_	*Z* = 2
*M**_r_* = 392.6	*F*(000) = 356
Triclinic, *P*1	*D*_x_ = 4.387 Mg m^−^^3^
*a* = 6.6123 (2) Å	Cu *K*α_1_ radiation, λ = 1.540562, 1.544390 Å
*b* = 8.7948 (3) Å	*T* = 293 K
*c* = 5.41074 (15) Å	Particle morphology: plate-like
α = 92.603 (2)°	white
β = 94.0843 (18)°	flat_sheet, 25 × 25 mm
γ = 71.3262 (18)°	Specimen preparation: Prepared at 1393 K and 100 kPa
*V* = 297.25 (2) Å^3^	

### Data collection

**Table:**

Panalytical XPert MPD Pro diffractometer	Data collection mode: reflection
Radiation source: sealed X-ray tube	Scan method: step
Specimen mounting: packed powder pellet	2θ_min_ = 8.013°, 2θ_max_ = 120.013°, 2θ_step_ = 0.017°

### Refinement

**Table:**

*R*_p_ = 0.024	67 parameters
*R*_wp_ = 0.032	0 restraints
*R*_exp_ = 0.015	6 constraints
*R*(*F*) = 0.024	Weighting scheme based on measured s.u.'s
6423 data points	(Δ/σ)_max_ = 0.005
Profile function: pseudo-Voigt	Background function: Legendre polynoms

### Fractional atomic coordinates and isotropic or equivalent isotropic displacement parameters (Å^2^)

**Table:**

	*x*	*y*	*z*	*U*_iso_*/*U*_eq_	
Na1	0.8823 (15)	0.0970 (12)	0.2630 (18)	0.035 (4)*	
Na2	0.3456 (14)	0.5024 (10)	0.7608 (18)	0.011 (3)*	
Hf1	0.2880 (3)	0.2710 (2)	0.2201 (3)	0.0158 (7)	
Si1	0.6528 (13)	0.1435 (9)	0.7769 (13)	0.010 (3)*	
Si2	0.9381 (12)	0.3380 (8)	0.6861 (15)	0.006 (2)*	
O1	0.307 (2)	0.0382 (17)	0.172 (3)	0.0030 (15)*	
O2	0.865 (2)	0.1777 (14)	0.727 (2)	0.0030 (15)*	
O3	0.494 (2)	0.2081 (15)	0.530 (3)	0.0030 (15)*	
O4	0.562 (2)	0.2522 (15)	0.020 (3)	0.0030 (15)*	
O5	−0.003 (2)	0.3116 (14)	0.401 (3)	0.0030 (15)*	
O6	0.120 (2)	0.3300 (14)	0.872 (3)	0.0030 (15)*	
O7	0.284 (2)	0.5148 (14)	0.288 (3)	0.0030 (15)*	

### Atomic displacement parameters (Å^2^)

**Table:**

	*U*^11^	*U*^22^	*U*^33^	*U*^12^	*U*^13^	*U*^23^
Hf1	0.0148 (10)	0.0177 (11)	0.0126 (10)	0.0003 (7)	0.0035 (7)	0.0111 (8)

### Geometric parameters (Å, º)

**Table:**

O1—Hf1	2.017 (15)	Na1—Na1^vii^	3.169 (13)
O1—Si1^i^	1.568 (17)	Na1—Na2^v^	3.363 (13)
O2—Si1	1.567 (18)	Na1—Hf1^viii^	3.513 (12)
O2—Si2	1.660 (17)	Na1—Si1^ii^	2.919 (12)
O3—Hf1	2.060 (13)	Na1—Si1	3.210 (13)
O3—Si1	1.644 (15)	Na1—Si1^vii^	3.140 (11)
O4—Na1	2.450 (15)	Na1—Si2	3.132 (13)
O4—Si1^ii^	1.622 (15)	Na2—Na2^v^	3.590 (14)
O5—Na1^iii^	2.323 (18)	Na2—Na2^ix^	3.174 (13)
O5—Si2^iii^	1.605 (16)	Na2—Hf1	3.556 (9)
O6—Hf1^iv^	2.115 (13)	Na2—Hf1^iv^	3.399 (10)
O6—Si2^iii^	1.497 (16)	Na2—Hf1^v^	3.590 (10)
O7—Na2^v^	2.441 (18)	Na2—Si1	3.161 (10)
O7—Si2^v^	1.625 (13)	Na2—Si2^v^	3.052 (11)
Na1—Na1^vi^	3.453 (14)		
			
Hf1—O1—Si1^i^	161.7 (9)	Na2^ix^—Na2—Si1	76.2 (3)
Si1—O2—Si2	136.8 (8)	Na2^ix^—Na2—Si2^v^	153.7 (4)
Hf1—O3—Si1	175.1 (10)	Hf1—Na2—Hf1^iv^	102.1 (3)
Na1—O4—Si1^ii^	89.2 (6)	Hf1—Na2—Hf1^v^	119.7 (3)
Na1^iii^—O5—Si2^iii^	104.3 (9)	Hf1—Na2—Si1	66.6 (2)
Hf1^iv^—O6—Si2^iii^	153.3 (10)	Hf1—Na2—Si2^v^	59.6 (2)
Na2^v^—O7—Si2^v^	134.4 (9)	Hf1^iv^—Na2—Hf1^v^	126.1 (3)
O4—Na1—O5^viii^	97.2 (6)	Hf1^iv^—Na2—Si1	62.6 (2)
O4—Na1—Na1^vi^	91.7 (4)	Hf1^iv^—Na2—Si2^v^	134.1 (4)
O4—Na1—Na1^vii^	152.6 (6)	Hf1^v^—Na2—Si1	102.9 (3)
O4—Na1—Na2^v^	51.1 (4)	Hf1^v^—Na2—Si2^v^	97.7 (3)
O4—Na1—Hf1^viii^	109.2 (5)	Si1—Na2—Si2^v^	125.7 (4)
O4—Na1—Si1^ii^	33.8 (4)	O1—Hf1—O3	88.5 (5)
O4—Na1—Si1	95.1 (5)	O1—Hf1—O6^ii^	91.8 (5)
O4—Na1—Si1^vii^	143.7 (5)	O1—Hf1—Na1^iii^	51.7 (4)
O4—Na1—Si2	103.7 (5)	O1—Hf1—Na2^ii^	123.8 (4)
O5^viii^—Na1—Na1^vi^	114.1 (6)	O1—Hf1—Na2	131.8 (4)
O5^viii^—Na1—Na1^vii^	89.9 (5)	O1—Hf1—Na2^v^	136.4 (4)
O5^viii^—Na1—Na2^v^	46.9 (4)	O3—Hf1—O6^ii^	171.1 (6)
O5^viii^—Na1—Hf1^viii^	36.4 (4)	O3—Hf1—Na1^iii^	107.6 (5)
O5^viii^—Na1—Si1^ii^	113.7 (5)	O3—Hf1—Na2^ii^	124.6 (5)
O5^viii^—Na1—Si1	85.7 (5)	O3—Hf1—Na2	50.0 (4)
O5^viii^—Na1—Si1^vii^	94.3 (5)	O3—Hf1—Na2^v^	71.4 (5)
O5^viii^—Na1—Si2	29.8 (4)	O6^ii^—Hf1—Na1^iii^	79.4 (4)
Na1^vi^—Na1—Na1^vii^	109.5 (3)	O6^ii^—Hf1—Na2^ii^	48.7 (4)
Na1^vi^—Na1—Na2^v^	116.9 (4)	O6^ii^—Hf1—Na2	133.2 (3)
Na1^vi^—Na1—Hf1^viii^	79.2 (3)	O6^ii^—Hf1—Na2^v^	102.6 (4)
Na1^vi^—Na1—Si1^ii^	58.3 (3)	Na1^iii^—Hf1—Na2^ii^	127.8 (2)
Na1^vi^—Na1—Si1	158.2 (4)	Na1^iii^—Hf1—Na2	111.9 (2)
Na1^vi^—Na1—Si1^vii^	52.3 (2)	Na1^iii^—Hf1—Na2^v^	171.0 (2)
Na1^vi^—Na1—Si2	141.2 (4)	Na2^ii^—Hf1—Na2	102.1 (2)
Na1^vii^—Na1—Na2^v^	125.8 (4)	Na2^ii^—Hf1—Na2^v^	53.9 (2)
Na1^vii^—Na1—Hf1^viii^	92.0 (3)	Na2—Hf1—Na2^v^	60.3 (2)
Na1^vii^—Na1—Si1^ii^	156.1 (5)	O1^i^—Si1—O2	111.9 (8)
Na1^vii^—Na1—Si1	59.0 (3)	O1^i^—Si1—O3	113.6 (9)
Na1^vii^—Na1—Si1^vii^	61.2 (3)	O1^i^—Si1—O4^iv^	109.9 (9)
Na1^vii^—Na1—Si2	70.6 (3)	O1^i^—Si1—Na1	97.9 (6)
Na2^v^—Na1—Hf1^viii^	71.7 (3)	O1^i^—Si1—Na1^iv^	76.2 (6)
Na2^v^—Na1—Si1^ii^	76.5 (3)	O1^i^—Si1—Na1^vii^	61.5 (6)
Na2^v^—Na1—Si1	83.1 (3)	O1^i^—Si1—Na2	150.4 (7)
Na2^v^—Na1—Si1^vii^	135.6 (5)	O2—Si1—O3	104.6 (8)
Na2^v^—Na1—Si2	55.9 (3)	O2—Si1—O4^iv^	105.7 (9)
Hf1^viii^—Na1—Si1^ii^	104.5 (4)	O2—Si1—Na1	52.6 (6)
Hf1^viii^—Na1—Si1	117.6 (3)	O2—Si1—Na1^iv^	77.6 (6)
Hf1^viii^—Na1—Si1^vii^	64.0 (3)	O2—Si1—Na1^vii^	50.5 (5)
Hf1^viii^—Na1—Si2	62.2 (3)	O2—Si1—Na2	97.4 (6)
Si1^ii^—Na1—Si1	123.9 (4)	O3—Si1—O4^iv^	110.8 (7)
Si1^ii^—Na1—Si1^vii^	110.6 (4)	O3—Si1—Na1	64.3 (6)
Si1^ii^—Na1—Si2	132.4 (4)	O3—Si1—Na1^iv^	167.4 (6)
Si1—Na1—Si1^vii^	120.1 (3)	O3—Si1—Na1^vii^	121.8 (6)
Si1—Na1—Si2	56.5 (3)	O3—Si1—Na2	59.9 (5)
Si1^vii^—Na1—Si2	103.3 (3)	O4^iv^—Si1—Na1	150.5 (8)
O7^v^—Na2—Na1^v^	99.3 (5)	O4^iv^—Si1—Na1^iv^	57.1 (5)
O7^v^—Na2—Na2^v^	45.5 (4)	O4^iv^—Si1—Na1^vii^	125.7 (6)
O7^v^—Na2—Na2^ix^	60.6 (4)	O4^iv^—Si1—Na2	55.6 (5)
O7^v^—Na2—Hf1	96.4 (4)	Na1—Si1—Na1^iv^	123.9 (4)
O7^v^—Na2—Hf1^iv^	113.5 (4)	Na1—Si1—Na1^vii^	59.9 (3)
O7^v^—Na2—Hf1^v^	35.8 (3)	Na1—Si1—Na2	103.3 (3)
O7^v^—Na2—Si1	68.4 (4)	Na1^iv^—Si1—Na1^vii^	69.4 (3)
O7^v^—Na2—Si2^v^	110.4 (5)	Na1^iv^—Si1—Na2	107.6 (3)
Na1^v^—Na2—Na2^v^	91.8 (3)	Na1^vii^—Si1—Na2	147.9 (4)
Na1^v^—Na2—Na2^ix^	97.3 (3)	O2—Si2—O5^viii^	100.7 (8)
Na1^v^—Na2—Hf1	117.5 (3)	O2—Si2—O6^viii^	106.7 (8)
Na1^v^—Na2—Hf1^iv^	124.7 (3)	O2—Si2—O7^v^	102.8 (8)
Na1^v^—Na2—Hf1^v^	64.7 (3)	O2—Si2—Na1	55.3 (5)
Na1^v^—Na2—Si1	167.6 (4)	O2—Si2—Na2^v^	103.7 (6)
Na1^v^—Na2—Si2^v^	58.2 (3)	O5^viii^—Si2—O6^viii^	116.1 (9)
Na2^v^—Na2—Na2^ix^	106.1 (3)	O5^viii^—Si2—O7^v^	109.8 (8)
Na2^v^—Na2—Hf1	60.3 (2)	O5^viii^—Si2—Na1	46.0 (6)
Na2^v^—Na2—Hf1^iv^	142.8 (3)	O5^viii^—Si2—Na2^v^	53.3 (5)
Na2^v^—Na2—Hf1^v^	59.4 (2)	O6^viii^—Si2—O7^v^	118.2 (8)
Na2^v^—Na2—Si1	80.2 (3)	O6^viii^—Si2—Na1	131.2 (6)
Na2^v^—Na2—Si2^v^	68.4 (3)	O6^viii^—Si2—Na2^v^	149.4 (8)
Na2^ix^—Na2—Hf1	141.7 (3)	O7^v^—Si2—Na1	110.3 (6)
Na2^ix^—Na2—Hf1^iv^	66.1 (3)	O7^v^—Si2—Na2^v^	57.1 (6)
Na2^ix^—Na2—Hf1^v^	60.0 (3)	Na1—Si2—Na2^v^	65.9 (3)

Symmetry codes: (i) −*x*+1, −*y*, −*z*+1; (ii) *x*, *y*, *z*−1; (iii) *x*−1, *y*, *z*; (iv) *x*, *y*, *z*+1; (v) −*x*+1, −*y*+1, −*z*+1; (vi) −*x*+2, −*y*, −*z*; (vii) −*x*+2, −*y*, −*z*+1; (viii) *x*+1, *y*, *z*; (ix) −*x*+1, −*y*+1, −*z*+2.
